# Complete Genome Sequence of Human Coronavirus Strain 229E Isolated from Plasma Collected from a Haitian Child in 2016

**DOI:** 10.1128/genomeA.01313-17

**Published:** 2017-11-22

**Authors:** Tania S. Bonny, Kuttichantran Subramaniam, Thomas B. Waltzek, Maha A. Elbadry, Valery Madsen Beau De Rochars, Taina Telisma, Mohammed Rashid, J. Glenn Morris, John A. Lednicky

**Affiliations:** a Department of Environmental and Global Health, College of Public Health and Health Professions, University of Florida, Gainesville, Florida, USA; b Emerging Pathogens Institute, University of Florida, Gainesville, Florida, USA; c Department of Infectious Diseases and Immunology, College of Veterinary Medicine, University of Florida, Gainesville, Florida, USA; d Department of Health Services Research, College of Public Health and Health Professions, University of Florida, Gainesville, Florida, USA; e Christianville Foundation School Clinic, Gressier, Haiti; f Department of Medicine, College of Medicine, University of Florida, Gainesville, Florida, USA

## Abstract

Human coronavirus strain 229E (HCoV-229E) and human alphaherpesvirus 1 were isolated from the plasma of a Haitian child in 2016 with suspected arbovirus diseases. To our knowledge, this is the first description of HCoV-229E in human plasma, which is the focus of this article.

## GENOME ANNOUNCEMENT

Human coronavirus strain 229E (HCoV-229E), one of the causative agents of the common cold, is increasingly associated with more severe respiratory infections in children, elders, and individuals with underlying medical conditions (1–4). The virus also has neuroinvasive and neurotropic properties (5). The presence of HCoV-229E within clinical samples (e.g., nasal or throat swabs, nasopharyngeal aspirate, bronchoalveolar lavage, or saliva) is typically confirmed by molecular (e.g., reverse transcription-PCR [RT-PCR] targeting of virus-specific genes) or serological (immunofluorescence assay targeting viral antigen) methods (3, 6–8). Primary isolation of HCoV-229E in cell culture is technically challenging, and few have succeeded at isolating the virus from respiratory specimens (3, 6, 9, 10). The isolation of HCoV-229E from human plasma, to our knowledge, has never been reported.

During a suspected arbovirus outbreak in March 2016, HCoV-229E and human alphaherpesvirus 1 (information to be presented elsewhere) were isolated from the plasma of a child presenting with acute undifferentiated febrile illness at a school clinic in rural Haiti. The study protocol was approved by the University of Florida Institutional Review Board (IRB) and the Haitian National IRB, with written consent obtained from parents/guardians of all study participants. The patient’s plasma tested negative for chikungunya, dengue, and zika genomic RNAs by RT-PCR (11). As the potential causative agent(s) was unknown, virus isolation was attempted in several human and animal cell lines. Virus-induced cytopathic effects (CPE) were observed in VERO E6, LLC-MK2, and MRC-5 by 14 days postinoculation of the cells. The CPE first consisted of either cell vacuolation or the formation of syncytia, followed by cell lysis or detachment of clumped cells from the cell monolayers, suggesting mixed infections. Infected cells and spent cell growth media were tested for the presence of viruses at various time points postinoculation using molecular assays. HCoV-229E viral genomic RNA (vRNA) was detected using a GenMark multiplex PCR eSensor XT-8 respiratory viral panel, and its identity was supported by the generation of correct-sized PCR amplicons using RT-PCR assays targeting HCoV-229E-specific genes (12–14). Whole-genome sequencing of HCoV-229E vRNA from Vero E6 cells was accomplished by Sanger sequencing following previously described methods (15) to attain the consensus sequence. In parallel, next-generation sequencing (NGS) with a cDNA library prepared using the NEBNext Ultra RNA library prep kit, and sequencing performed using a version 3 chemistry 600-cycle kit on a MiSeq platform (Illumina), produced the same consensus sequence.

Whole-genome sequence analyses of the Haitian HCoV-229E isolate, designated strain 229E/Haiti-1/2016, revealed close genetic relatedness (>99% nucleotide identity) to several American HCoV-229E strains reported in 2015 (the GenBank accession numbers of the three HCoV-229E sequences with the highest nucleotide similarities as of 22 July 2017 are KY983587, KY684760, and KY967357). Compared to the American strains, the Haitian HCoV-229E genome has unique nucleotide polymorphisms that result in changes within the deduced amino acid sequences of the replicase polyprotein 1ab, spike, accessory, membrane, envelope, and nucleocapsid proteins.

### Accession number(s).

The complete genome sequence of HCoV-229E strain 229E/Haiti-1/2016 has been deposited in the GenBank database under accession number MF542265.

## References

[1] van der Hoek L 2007 Human coronaviruses: what do they cause? Antivir Ther 12:651–658. 17944272

[2] Walsh EE, Shin JH, Falsey AR 2013 Clinical impact of human coronaviruses 229E and OC43 infection in diverse adult populations. J Infect Dis 208:1634–1642. doi:10.1093/infdis/jit393. 23922367 PMC3805243

[3] Pene F, Merlat A, Vabret A, Rozenberg F, Buzyn A, Dreyfus F, Cariou A, Freymuth F, Lebon P 2003 Coronavirus 229E-related pneumonia in immunocompromised patients. Clin Infect Dis 37:929–932. doi:10.1086/377612. 13130404 PMC7107892

[4] Gorse GJ, O’Connor TZ, Hall SL, Vitale JN, Nichol KL 2009 Human coronavirus and acute respiratory illness in older adults with chronic obstructive pulmonary disease. J Infect Dis 199:847–857. doi:10.1086/597122. 19239338 PMC7110218

[5] Desforges M, Le Coupanec A, Stodola JK, Meessen-Pinard M, Talbot PJ 2014 Human coronaviruses: viral and cellular factors involved in neuroinvasiveness and neuropathogenesis. Virus Res 194:145–158. doi:10.1016/j.virusres.2014.09.011. 25281913 PMC7114389

[6] Farsani SM, Dijkman R, Jebbink MF, Goossens H, Ieven M, Deijs M, Molenkamp R, van der Hoek L 2012 The first complete genome sequences of clinical isolates of human coronavirus 229E. Virus Genes 45:433–439. doi:10.1007/s11262-012-0807-9. 22926811 PMC7088690

[7] Chibo D, Birch C 2006 Analysis of human coronavirus 229E spike and nucleoprotein genes demonstrates genetic drift between chronologically distinct strains. J Gen Virol 87:1203–1208. doi:10.1099/vir.0.81662-0. 16603522

[8] Kim YG, Yun SG, Kim MY, Park K, Cho CH, Yoon SY, Nam MH, Lee CK, Cho YJ, Lim CS 2017 Comparison between saliva and nasopharyngeal swab specimens for detection of respiratory viruses by multiplex reverse transcription-PCR. J Clin Microbiol 55:226–233. doi:10.1128/JCM.01704-16. 27807150 PMC5228234

[9] Dijkman R, Jebbink MF, Koekkoek SM, Deijs M, Jónsdóttir HR, Molenkamp R, Ieven M, Goossens H, Thiel V, van der Hoek L 2013 Isolation and characterization of current human coronavirus strains in primary human epithelial cell cultures reveal differences in target cell tropism. J Virol 87:6081–6090. doi:10.1128/JVI.03368-12. 23427150 PMC3648119

[10] Matoba Y, Aoki Y, Tanaka S, Yahagi K, Itagaki T, Matsuzaki Y, Mizuta K 2015 Picornavirus-like cytopathic effects on RD-18S cell lines were induced by human coronavirus 229E not picornaviruses. Jpn J Infect Dis 68:78–79. doi:10.7883/yoken.JJID.2014.487. 25702793

[11] Waggoner JJ, Gresh L, Mohamed-Hadley A, Ballesteros G, Davila MJ, Tellez Y, Sahoo MK, Balmaseda A, Harris E, Pinsky BA 2016 Single-reaction multiplex reverse transcription PCR for detection of Zika, chikungunya, and dengue viruses. Emerg Infect Dis 22:1295–1297. doi:10.3201/eid2207.160326. 27184629 PMC4918162

[12] Vabret A, Mouthon F, Mourez T, Gouarin S, Petitjean J, Freymuth F 2001 Direct diagnosis of human respiratory coronaviruses 229E and OC43 by the polymerase chain reaction. J Virol Methods 97:59–66. doi:10.1016/S0166-0934(01)00343-3. 11483217 PMC7119936

[13] Myint S, Johnston S, Sanderson G, Simpson H 1994 Evaluation of nested polymerase chain methods for the detection of human coronaviruses 229E and OC43. Mol Cell Probes 8:357–364. doi:10.1006/mcpr.1994.1052. 7877631 PMC7135046

[14] Stewart JN, Mounir S, Talbot PJ 1992 Detection of coronaviruses by the polymerase chain reaction, p 316–327. *In* Becker Y, Darai G (ed) Diagnosis of human viruses by polymerase chain reaction technology, frontiers of virology, vol 1. Springer, Berlin, Germany.

[15] Bonny TS, Yezli S, Lednicky JA 8 9 2017 Isolation and identification of *human coronavirus 229E* from frequently touched environmental surfaces of a university classroom that is cleaned daily. Am J Infect Control. doi:10.1016/j.ajic.2017.07.014. PMC7115338 28893443

